# 

**Published:** 1897-08

**Authors:** 


### CONTENTS:

### SELECTIONS.

Article I.—The Patient. By Chas. T. Howard, D.D.S. .......... 145
" II.—Economy in Dental Practice. By R. E. Sparks, D.D.S. 149
" III.—Plantation of Teeth. By Dr. Louis Jack .......... 155
" IV.—Separating Teeth .......... 163
" V.—The Study of Anatomy. By W. C. Barrett, M. D., D. D. S., M. D. S. .......... 168
" VI.—How and Why we Treat and Fill Root-Canals—Our Way. By W. A. Mills, D D. S .......... 175

### COMMUNICATED.

National Association of Dental Faculties .......... 177
National Association of Dental Examiners .......... 185

### OBITUARY.

Dr. Samuel J. Hayes .......... 185

### MONTHLY SUMMARY.

Anti-Extraction .......... 187

Life and Death in Emotions .......... 188

Improved Poreclain Bridge .......... 189

Tuberculosis of the Alveolar Process .......... 190

An Interesting Case of Membranous Stomatitis .......... 190

No Sympathy for Non-contributors .......... 191

Adhæsol .......... 191

Incisor or Cuspid Crown .......... 192

Teeth in Relation to the Ear, Nose and Troat .......... 192

Hearing Restored after Twenty-five Years .......... 192

